# Deep Cavitand Calixarene–Solubilized Fullerene as a Potential Photodynamic Agent

**DOI:** 10.3389/fchem.2021.710808

**Published:** 2021-06-29

**Authors:** Tian-Xing Zhang, Juan-Juan Li, Hua-Bin Li, Dong-Sheng Guo

**Affiliations:** College of Chemistry, Key Laboratory of Functional Polymer Materials (Ministry of Education), State Key Laboratory of Elemento-Organic Chemistry, Tianjin Key Laboratory of Biosensing and Molecular Recognition, Nankai University, Tianjin, China

**Keywords:** supramolecular chemistry, photodynamic agent, calixarene, fullerene, solubility

## Abstract

Fullerene has attracted much attention in biomedical research due to its unique physical and chemical properties. However, the hydrophobic nature of fullerene is limited to deploy in the body, given that the biofluids are mainly water. In this study, a water-soluble supramolecular nanoformulation based on a deep cavitand calixarene (SAC4A) and fullerene is developed to overcome the hydrophobicity of fullerene and is used as a potential photodynamic agent. SAC4A solubilizes fullerene very well with a simple grinding method. The significantly increased water solubility of fullerene enables efficient activation of reactive oxygen species. The host–guest strategy to solubilize fullerene can not only provide a new method to achieve water solubility but also expand the biomedical applications of fullerene.

## Introduction

Fullerene has been widely used in biomedical research, acting as an antimicrobial agent (Mashino et al., 1999; Tsao et al., 2001), a human immunodeficiency virus protease inhibitor (Friedman et al., 1993), and a photosensitizer to cleave DNA (Boutorine et al., 1995; Sharma et al., 2011). It can efficiently form long-lived triplet excited states by visible-light irradiation and generate highly reactive oxygen species (ROS) *via* an electron transfer Type I reaction, which generates superoxide anions (O_2_^−•^) yielding hydroxyl radicals, and/or an energy transfer Type II reaction, which generates singlet oxygen molecules (^1^O_2_) (Yamakoshi et al., 2003). However, the hydrophobicity of fullerene limits its potential applications as photosensitizer in biological fluids. Much effort has been focused on increasing fullerene water solubility by grafting hydrophilic groups on fullerene (Rašović, 2016). Nevertheless, chemical modifications usually lead to the unanticipated alternation of fullerene photophysical properties (Hamano et al., 1997; Prat et al., 1999). Therefore, the solubilization of fullerene in a non-covalent approach emerges to be an alternative approach (Zhang et al., 2014). Macrocyclic hosts have been engaged in solubilizing fullerene in water [e.g., cyclodextrins (CD) and calixarenes] (Ikeda, 2013). Braun et al. investigated the solid–solid mechanochemical reaction between fullerene and γ-CD by ball-milling their mixture. The concentration of C_60_ in water was 1.5 × 10^–4^ M [(γ-CD) = 6.5 × 10^–3^ M] (Braun et al., 1994). Komatsu et al. examined that the equimolar amounts of C_60_ and sulfonatocalix[8]arene were subjected to high-speed vibration milling treatment, and the concentration of C_60_ was calculated to be 1.3 × 10^–4^ M (Komatsu et al., 1999). These water-soluble fullerenes solubilized by macrocycles confirm the feasibility of the host–guest strategy. Moreover, the complexation of fullerenes by macrocycles is an important way used to improve their photoactivities (Antoku et al., 2019).

**FIGURE 1:**
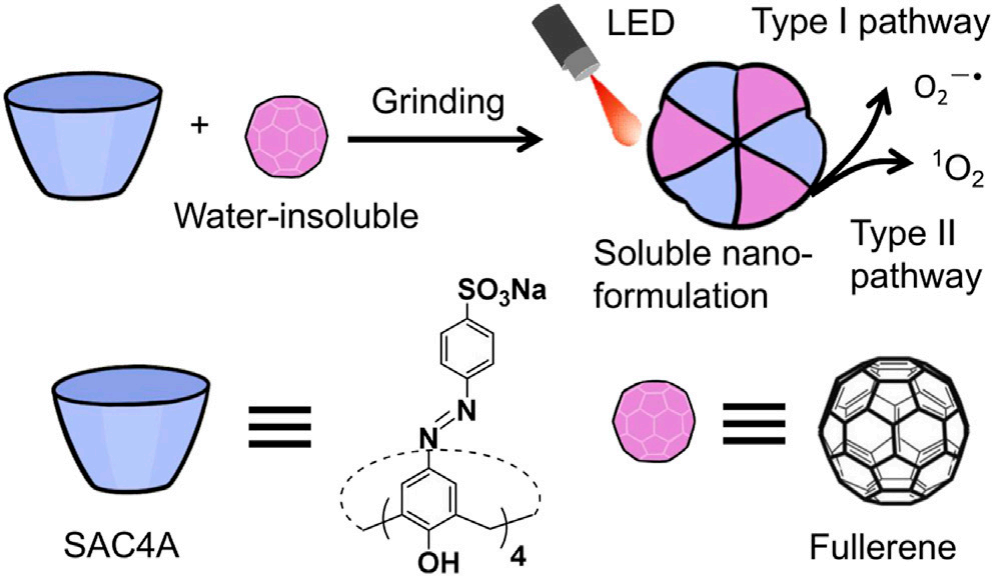
Schematic illustration of supramolecular nanoformulation formed by the host–guest complexation between SAC4A and fullerene. The encapsulation of fullerene with SAC4A gives rise to improved solubility and efficiently activated ROS.

In this work, we synthesized sulfonated azocalix[4]arene (SAC4A), which possesses a deep cavity that imparts strong binding to hydrophobic cargoes. Additionally, the -SO_3_^−^ functional groups endow calixarene with water solubility and also provide anchoring points that supplement the cavity binding to guests (Guo et al., 2014; Pan et al., 2017; Pan et al., 2021; Pan et al., 2021). As expected, SAC4A solubilized fullerene under the condition of a molar ratio of 1:1 by the grinding method (Figure 1), which is a simpler method than ball-milling or high-speed vibration milling treatment. We further evaluated its ability to generate ROS under LED irradiation, which is significant on account of its further biomedical applications.

## Results and Discussion

### Molecular Design of Macrocyclic Host SAC4A and SAC4A-Solubilized Fullerene

**FIGURE 2:**
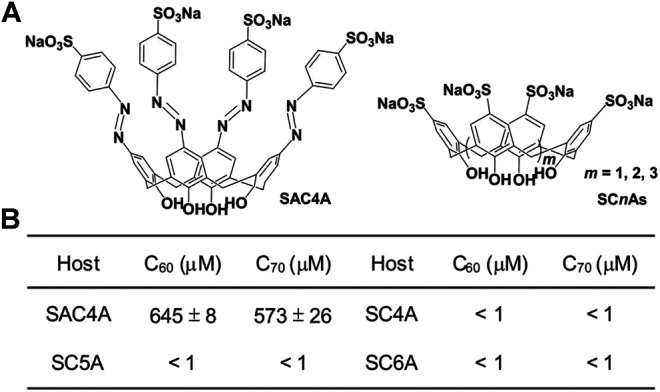
**(A)** Molecular structures of SAC4A and SC*n*As. **(B)** The concentrations of C_60_ and C_70_ solubilized by calixarenes (1 mM for hosts).

Calixarene was employed as the macrocyclic host because of its broad chemical design space (Böhmer, 1995). By making calix[4]arene to directly react with 4-sulfobenzenediazonium chloride, SAC4A was obtained with a high yield (Figure 2A and Supplementary Figure S1) (Lu et al., 2005). Sulfocalix[*n*]arenes (SC*n*As, *n* = 4, 5, and 6) without deep cavity were synthesized as controls (Figure 2A), referring to literatures (Shinkai et al., 1987; Steed et al., 1995). The calixarene-solubilized fullerenes (C_60_ and C_70_) were prepared by the grinding method (for more details, see the Supplementary Material). The concentrations of C_60_ and C_70_ in the supernatant were determined by high-performance liquid chromatography (Figure 2B). The concentration of C_60_ by supramolecular complexation with SAC4A increased to 6.45 × 10^–4^ M. The solubilization effect of SAC4A is significantly higher than that of the control of SC*n*As. It may be observed that the cavity of SC4A is too small to accommodate fullerene. Even if adding repetitive units to expand the cavity latitudinally, the cavities of SC5A and SC6A are still too shallow to encapsulate fullerene. On the other hand, the azobenzene modification endows SAC4A with a deep cavity that is suitable to include hydrophobic guests (Zhang et al., 2020). It is suggested that calixarene is more effective in solubilizing fullerene by extending the cavity longitudinally than latitudinally.

### Size, Morphology, and Stability of C_60_@SAC4A

**FIGURE 3:**
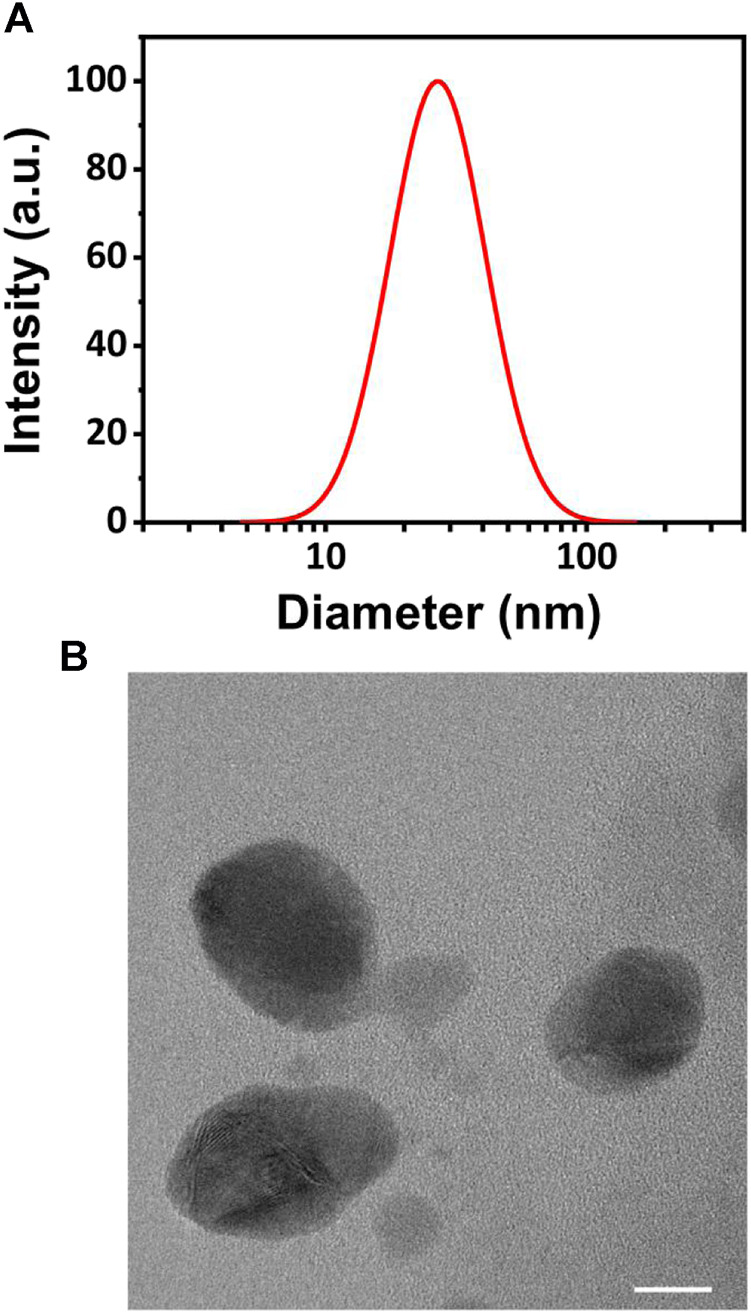
**(A)** The DLS data of the C_60_@SAC4A assembly in PBS buffer (10 mM, pH = 7.4), at 25°C. **(B)** TEM image of the C_60_@SAC4A assembly (scale bar, 10 nm).

Due to the best solubilizing effect, we selected the C_60_@SAC4A system to representatively study its size and morphology. Dynamic light scattering (DLS) was employed to identify the size of the C_60_@SAC4A assembly (Guo et al., 2020), giving a hydration diameter of 28 nm with a polydispersity index (PDI) of 0.135 (Figure 3A). The representative transmission electron microscopy (TEM) image (Li et al., 2020) revealed that the C_60_@SAC4A assembly possessed a spherical morphology with an average size of 20 nm (Figure 3B), which was smaller than the DLS result due to the dehydration that occurred during the TEM sample preparation (Yang et al., 2020). C_60_@SAC4A forms water-soluble nanoformulation rather than simple host–guest complex, implying a potential passive targeting ability through enhanced permeation and retention (EPR) effect when used *in vivo* (Gao et al., 2018; Cai et al., 2021).

The stability of assembly is a fundamental factor. A DLS test was performed to observe the particle size changes of C_60_@SAC4A at different time points (Supplementary Figure S2). The C_60_@SAC4A assembly shows no significant changes in particle size within 7 days. These results proved that the C_60_@SAC4A assembly is stable in time. SAC4A is expected to be a supramolecular solubilizing agent to disperse fullerene in water.

### ROS Generation of C_60_@SAC4A

**FIGURE 4:**
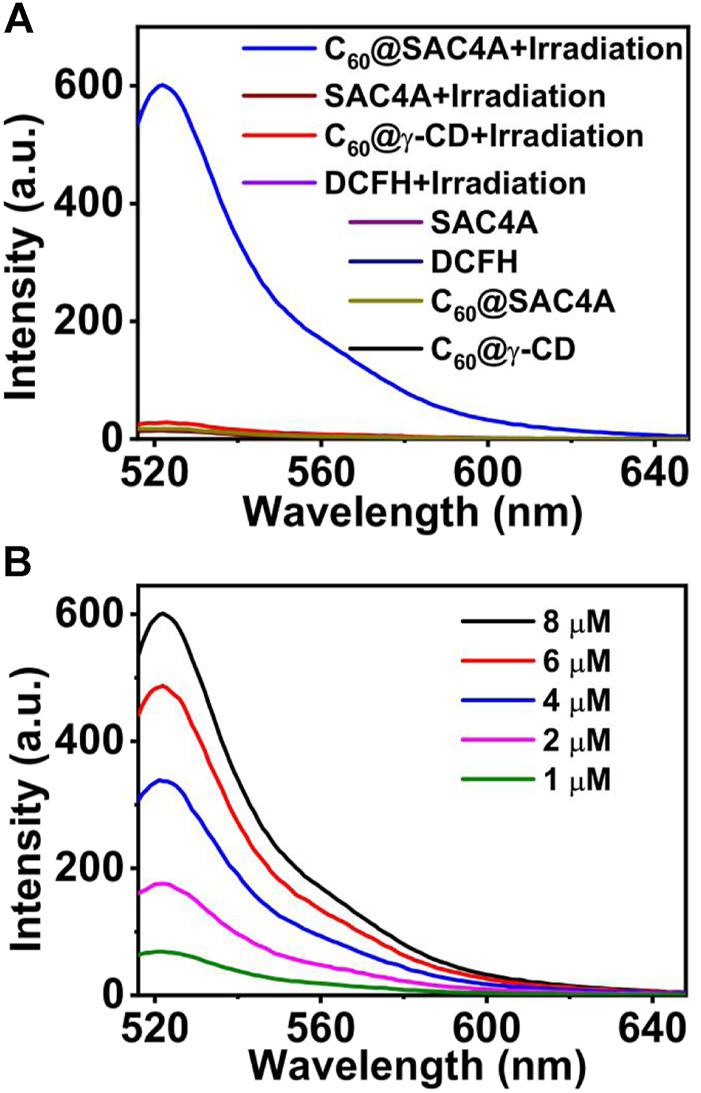
**(A)** Fluorescence response of DCFH to ROS treated with C_60_@SAC4A (8 μM for C_60_), SAC4A (10 μM), and C_60_@γ-CD (8 μM for C_60_) under LED irradiation for 1 min or no irradiation. **(B)** Fluorescence response of DCFH to ROS treated with C_60_@SAC4A of different concentrations under LED irradiation for 1 min.

The identification and research of ROS are crucial for the further development of photodynamic therapy based on fullerenes as photosensitizers. In order to see if it was a potential photodynamic agent, the ROS generation capacities of C_60_@SAC4A were investigated by a commercial indicator, 2,7-dichlorodihydrofluorescein (DCFH), that responds to general types of ROS (Zhuang et al., 2020). Upon exposure to LED irradiation, the nonemissive DCFH solution with C_60_@SAC4A exhibits increased fluorescence intensity, accomplishing approximately 35-fold enhancement within 1 min, while those of control groups under the same conditions are barely increased (Figure 4A). Additionally, γ-cyclodextrin–solubilized C_60_ (C_60_@γ-CD) was chosen as the positive control group, showing a little fluorescence increase under LED irradiation, due to the reactivity of C_60_ with O_2_ decreased when it was enclosed in the γ-CD cavity (Priyadarsini et al., 1994). Another possible reason is that the amount of cyclodextrin used for solubilization is much higher than that of calixarene, resulting that the cyclodextrin limits the contact between C_60_ and O_2_. Figure 4B showed the fluorescence response of DCFH to ROS treated with C_60_@SAC4A of different concentrations, suggesting that the peak intensity and the concentration of C_60_@SAC4A were increased in a concentration-dependent manner.

**FIGURE 5:**
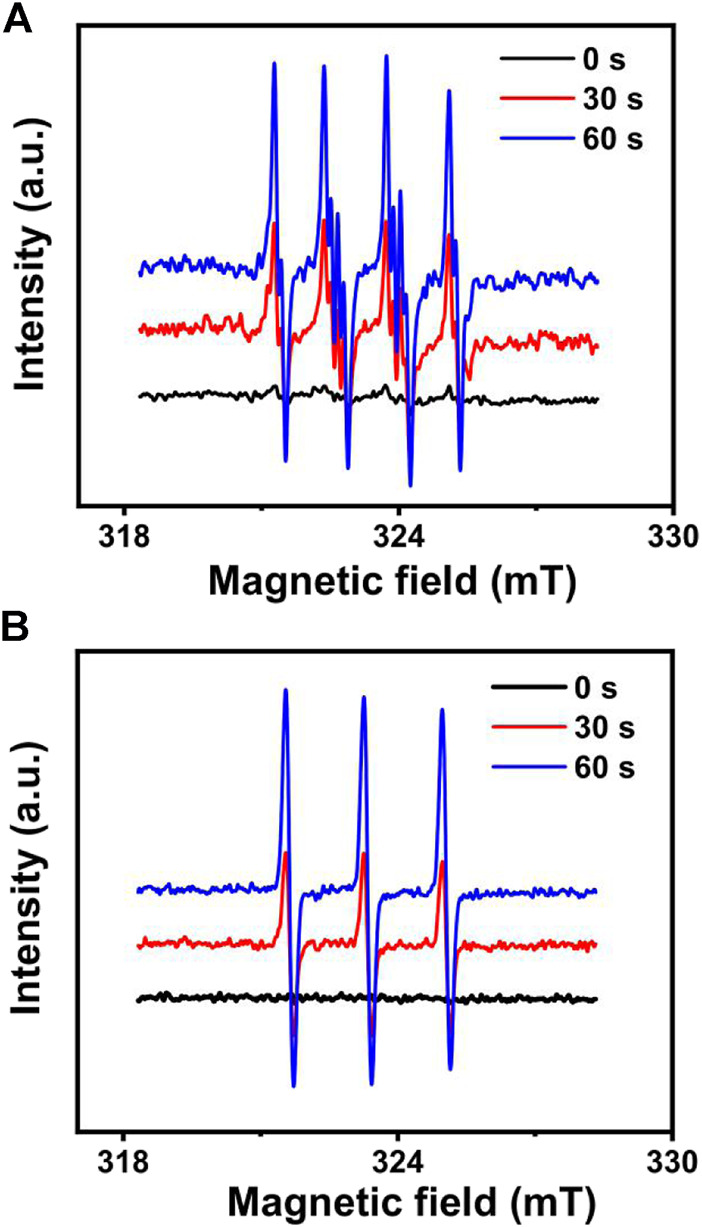
**(A)** EPR signals of DMPO (for Type I ROS detection) in the presence of C_60_@SAC4A (80 μM for C_60_) with NADH (5 mM) irradiated for 0, 30, or 60 s. **(B)** EPR signals of TEMP (for Type II ROS detection) in the presence of C_60_@SAC4A (80 μM for C_60_) irradiated for 0, 30, or 60 s.

Electron paramagnetic resonance (EPR) spectroscopy is one powerful method to identify short-lived ROS species (Zang et al., 1998). 5,5-Dimethyl-1-pyrroline-*N*-oxide (DMPO) was used as a spin-trap agent to detect O_2_^−•^. Figure 5A shows the spectra obtained for DMPO/O_2_^−•^ adduct, indicative of the generation of O_2_^−•^ from C_60_@SAC4A under light and nicotinamide adenine dinucleotide (reduced, NADH, to mimic the strong reducing environment) conditions, namely, Type I ROS (Zhao et al., 2008). In the absence of either NADH or C_60_, there were no appreciable O_2_^−•^ signals detected (Supplementary Figure S3A), showing that electron transfer processes induced by reducing agents are very significant for the generation of O_2_^−•^ in aqueous systems (Yamakoshi et al., 2003). For the detection of ^1^O_2_, the EPR method with 2,2,6,6-tetramethyl-4-piperidone (TEMP) was employed. As shown in Figure 5B, the resultant EPR spectra displayed a typical 1:1:1 triplet signal, which is the characteristic resonance for TEMP/^1^O_2_ adduct, whereas no signal was detected in the control group containing SAC4A under the same condition (Supplementary Figure S3B), indicative of its good ability of Type II ROS (Yamakoshi et al., 2003). Based on the above evidence, it is consequently reasonable to draw a conclusion that C_60_@SAC4A followed both Type I and Type II pathways to generate ROS species, indicating that C_60_@SAC4A can be a promising candidate as a water-soluble supramolecular photosensitizer.

## Conclusion

In summary, SAC4A was synthesized and used to improve the water solubility of fullerene by host–guest complexation. Compared with SC*n*As that are widely studied as classical water-soluble calixarene derivatives, SAC4A possesses the deeper cavity longitudinally and is more effective in solubilizing fullerene. The supramolecular nanoformulation C_60_@SAC4A generates ROS species effectively in both Type I and Type II pathways, indicative of a potential photodynamic agent. Calixarene is highly modifiable, and thus, a lot of water-soluble derivatives could be obtained to solubilize hydrophobic substances besides fullerenes. One important lesson from this work is that vertical expansion of cavity emerges to be a more powerful way than horizontal expansion to solubilize large hydrophobic species on account of the cone shape of calixarene.

## Data Availability

The original contributions presented in the study are included in the article/Supplementary Material; further inquiries can be directed to the corresponding authors.
