# Supplementary material for: Deep Cavitand Calixarene–Solubilized Fullerene as a Potential Photodynamic Agent
Source: Front Chem. 2021 Jun 29;9:710808. doi: 10.3389/fchem.2021.710808 (PMC8327297; doi:10.3389/fchem.2021.710808)
Supplement: Supplementary file 1 [file datasheet1.docx]

Supplementary Material

for

Deep Cavitand Calixarene–Solubilized Fullerene as a Potential Photodynamic Agent

Tian-Xing Zhang^†^, Juan-Juan Li^†^, Hua-Bin Li^*^ and Dong-Sheng Guo^*^

College of Chemistry, Key Laboratory of Functional Polymer Materials (Ministry of Education), State Key Laboratory of Elemento-Organic Chemistry, Tianjin Key Laboratory of Biosensing and Molecular Recognition, Nankai University, Tianjin 300071, China

*** Correspondence:**Hua-Bin Li
lihuabin@nankai.edu.cn

Dong-Sheng Guo
dshguo@nankai.edu.cn

# Materials and Apparatus

All the reagents and solvents were commercially available and used as received unless otherwise specified purification. C_60_, C_70_ and 2,7-dichlorofluorescein diacetate (DCFH-DA) were purchased from Aladdin. Diethylenetriaminepentaacetic acid (DETAPAC), 2,2,6,6-tetramethyl-4-piperidone (TEMP) and γ-cyclodextrin (γ-CD) were obtained from J&K Chemical. Nicotinamide adenine dinucleotide (reduced, NADH) was purchased from TCI. The phosphate buffered saline (PBS, 10 mM) solution of pH = 7.4 and 5,5-Dimethyl-1-pyrroline-*N*-oxide (DMPO) were purchased from Macklin.

NMR data were recorded on a Bruker AV400 spectrometer. Steady-state fluorescence measurements were recorded in a conventional quartz cell (light path 10 mm) on a Cary Eclipse equipped with a Cary single-cell peltier accessory. Mass spectra were performed on an Autoflex III TOF/TOF200 (MALDI-TOF). High-performance liquid chromatography (HPLC) system (Waters e2695, Milford, MA, USA) was employed to perform chromatographic analysis. The TEM sample was examined by a high-resolution TEM (Tecnai G2 F20 microscope, FEI) equipped with a CCD camera (Orius 832, Gatan). The sample solutions for dynamic light scattering (DLS) measurements were examined on a laser light scattering spectrometer (NanoBrook 173plus). EPR spectra were recorded using a JES-FA200 spectrometer (JEOL).

# Syntheses of SAC4A and SC*n*As

**Scheme S1**. The synthetic route of SAC4A.

**5,11,17,23-Tetrakis[(*p*-sulfophenyl)azo]-25,26,27,28-tetrahydroxycalix[4]arene** (SAC4A) (Lu et al, 2005). Sulfanilic acid (1.732 g, 10 mmol) was dissolved in water (10 mL) containing sodium carbonate of (0.518 g, 5 mmol) at 50-55 °C. A solution of NaNO_2_ (0.702 g, 10 mmol) in water (10 mL) was added to sulfanilic acid solution and then this mixture was added slowly to the concentrated HCl (4 mL) at 0-5 °C for 30 min and further stirred at this temperature for 1.5 h to get 4-sulfobenzenediazonium chloride salt. The obtained solution was slowly added into a solution of 25,26,27,28-tetrahydroxycalix[4]arene (C4A, 1.000 g, 2.36 mmol) and sodium acetate trihydrate (4.080 g, 30 mmol) in MeOH-DMF (26 mL, 5:8, v:v) to obtain a red suspension. The red reactant was allowed to couple for 2 h more in an ice bath, then acidified by 150 mL of aqueous HCl (0.25%) and warmed at 60 °C for 30 min to produce reagent in a quantitative yield as a reddish viscous solid. The solvent was evaporated to dryness, the treatment of the residue with chloroform gave a precipitate. After filtered out it was dissolved by water, the insoluble solid was removed and a new precipitate performed when methanol was added, then the pure product was obtained by filtration (2.467 g, 84%). ^1^H NMR (400 MHz, DMSO-*d_6_*) *δ* 7.77 (s, 8H, calix-Ar-H), 7.69 (m, 16H, Ar-H), 4.39 and 3.66 (s, 8H, Ar-CH_2_-Ar) ppm; Mass spectrum (MALDI-TOF): [M+Na]^+^: *m/z* calcd. for (C_52_H_36_N_8_Na_5_O_16_S_4_^+^): 1271.0621, found: 1271.0618.

Sulfonatocalix[*n*]arenes (SC*n*As, *n* = 4, 5, 6) were synthesized according to previous literatures (Shinkaiet et al, 1987; Steed et al., 1995).

# General Methods

## Solubility Enhancements

Briefly, 0.006 mmol of C_60_ or C_70_ was mixed with 1 equiv. of SAC4A or SC*n*As (or 10 equiv. of γ-CD) in a mortar. The mixture was ground for 30 min and was collected with 6 mL PBS in a bottle. The suspension was shaken for 2 h (200 rpm, 25 °C) and then centrifuged at 10000 rpm for 10 min. Filter the supernatant with 0.22 μm water membrane and the concentration of C_60_ or C_70_ in the supernatant obtained again was determined by HPLC. Carried on HT-ODS-100-5-P column (4.6 mm × 250 mm) at 25 °C, the chromatographic separation was accomplished by mobile phase consisting of methanol and toluene (1:1) in an isocratic elution. The detection wavelength was 310 nm. The flow rate of mobile phase was set at 1 mL min^−1^ and the injection volume was 10 μL. The solubility enhancement assay was performed in triplicate.

## Size, Morphology, and Stability Evaluations

C_60_@SAC4A (8 μM for C_60_) in PBS was detected for the size measurement by DLS. Then, the hydrodynamic size of C_60_@SAC4A was detected at 25 °C for 7 days to further evaluate the stability. TEM grids were prepared by evaporating 20 μL of C_60_@SAC4A (20 μM for C_60_) onto a copper grid.

## ROS Detections

General ROS detections by fluorescence analysis. The general ROS generation measurements were conducted using 2,7-dichlorodihydrofluorescein (DCFH) as the indicator (Zhuang et al., 2020), which was converted from 2,7-dichlorodihydrofluorescein diacetate (DCFH-DA, 0.5 mL, 1 mM in ethanol) reacting with aqueous solution of NaOH (2 mL, 1.0 mM) for 30 min at room temperature. The hydrolysate was then neutralized with 7.5 mL PBS buffer solution to get the stock solution with a concentration of 50 μM. PBS buffer solution containing 1 μM DCFH was added C_60_@SAC4A (8 μM for C_60_), SAC4A (10 μM), and C_60_@γ-CD (8 μM for C_60_). The fluorescence signal of indicator was monitored in a range of 510–650 nm with the excitation wavelength at 504 nm after the solution was irradiated by white LED of 228 mW cm^−2^ for 1 min.

ROS detections by EPR analysis. EPR analysis was performed to monitor the generation of Type I ROS using 5,5-Dimethyl-1-pyrroline-*N*-oxide (DMPO) as spin-trap agent (Zhao et al., 2008). PBS buffer solution containing DMPO (80 mM), DETAPAC (1 mM), DMSO (3.1 M) was added C_60_@SAC4A (80 μM) without/with NADH (5 mM), SAC4A (80 μM) with NADH (5 mM). Spectra of spin were monitored after the solutions were irradiated by a 1 kW Xe arc-lamp (*λ* > 400 nm). Background interference was corrected using the sample before irradiation. EPR analysis was performed to monitor the generation of Type II ROS using 2,2,6,6-tetramethyl-4-piperidone (TEMP) as spin-trap agent (Yamakoshi et al., 2003). D_2_O containing 80 mM TEMP was added C_60_@SAC4A (80 μM), SAC4A (80 μM). Spectra of spin were monitored after the solutions were irradiated by a 1 kW Xe arc-lamp (*λ* > 400 nm). Background interference was corrected using the sample before irradiation.

a


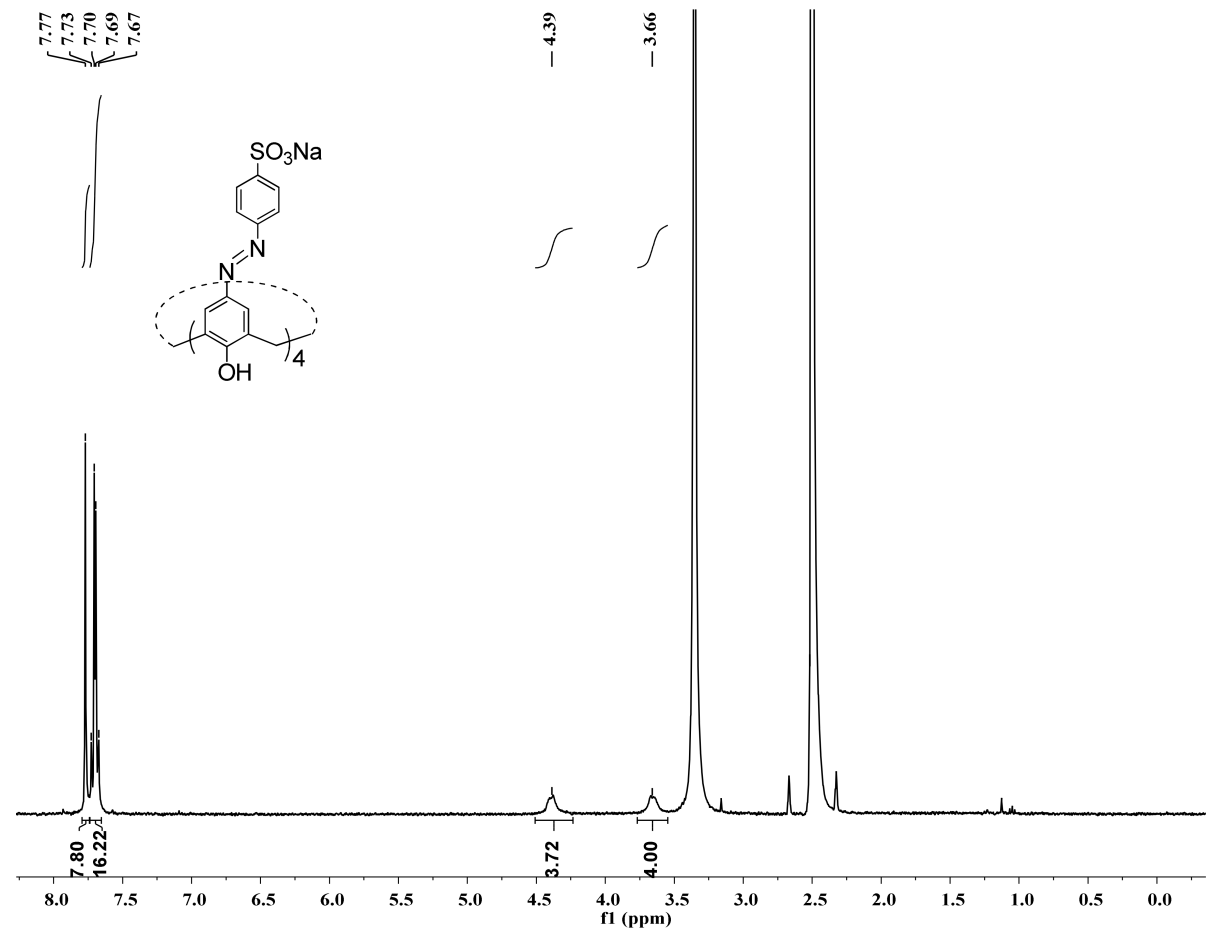


b


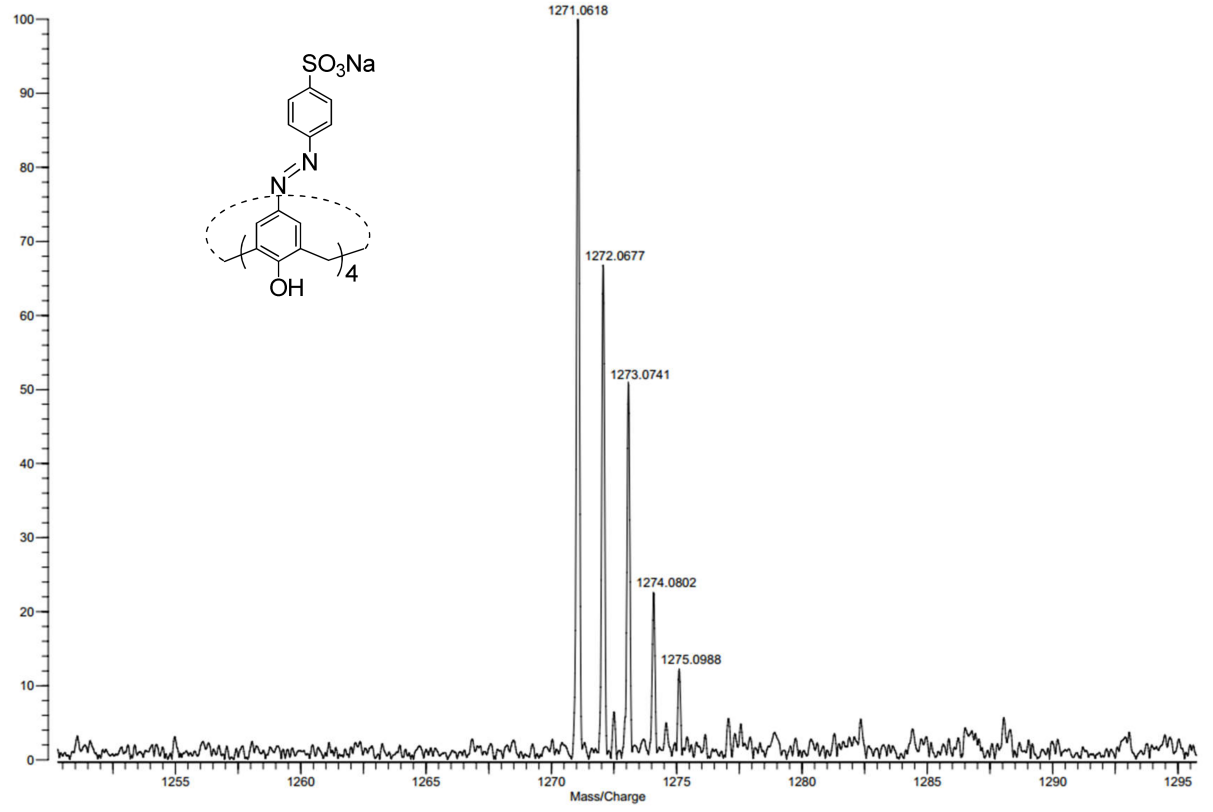


**Supplementary Figure S1.** (a) ^1^H NMR spectrum of SAC4A in DMSO-*d_6_*, 400 MHz, 25 °C. (b) Mass spectrum (MALDI-TOF) of SAC4A.


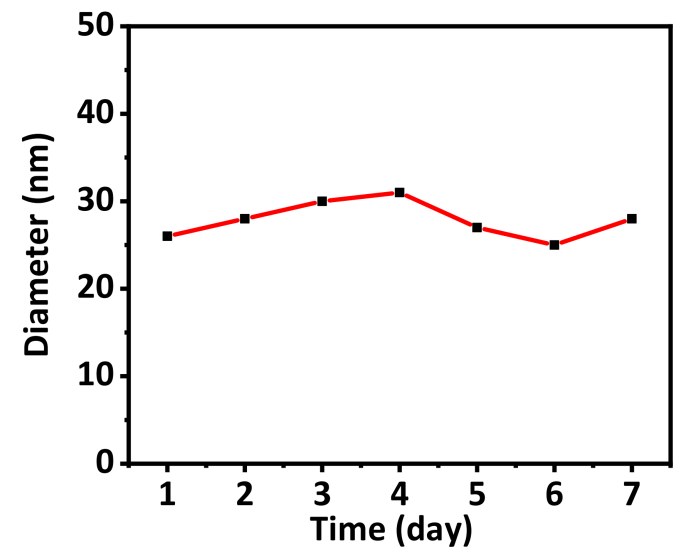


**Supplementary Figure S2.** The size change of C_60_@SAC4A as a function of time for 7 days. Experimental conditions: PBS buffer (10 mM, pH = 7.4), 25 °C.


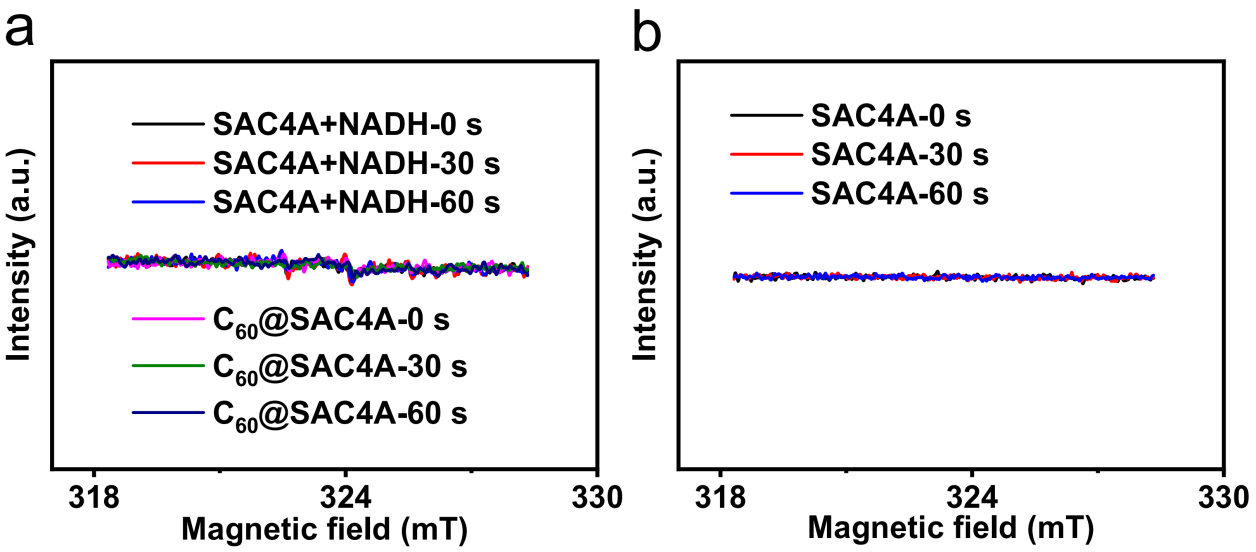


**Supplementary Figure S3.** (a) EPR signals of DMPO (for Type I ROS detection) in the presence of SAC4A (80 μM) with NADH (5 mM), C_60_@SAC4A (80 μM for C_60_) irradiated for 0 s, 30 s or 60 s. (b) EPR signals of TEMP (for Type II ROS detection) in the presence of SAC4A (80 μM) irradiated for 0 s, 30 s or 60 s.

# References

Lu, L., Zhu, S., Liu, X., Xie, Z., and Yan, X. (2005). Highly selective chromogenic ionophores for the recognition of chromium(III) based on a water-soluble azocalixarene derivative. *Anal. Chim. Acta* 535, 183–187. doi: 10.1016/j.aca.2004.11.059

Shinkai, S., Araki, K., Tsubaki, T., Arimura, T., and Manabe, O. (1987). New syntheses of calixarene-*p*-sulphonates and *p*-nitrocalixarenes. *J. Chem. Soc. Perkin Trans.* 1, 2297–2299. doi: 10.1039/P19870002297

Steed, J. W., Johnson, C. P., Barnes, C. L., Juneja, R. K., Atwood, J. L., Reilly, S., et al. (1995). Supramolecular chemistry of *p*-Sulfonatocalix[5]arene: a water-soluble, bowl-shaped host with a large molecular cavity. *J. Am. Chem. Soc.* 117, 11426–11433. doi: 10.1021/ja00151a006

Yamakoshi, Y., Umezawa, N., Ryu, A., Arakane, K., Miyata, N., Goda, Y., et al. (2003). Active oxygen species generated from photoexcited fullerene (C_60_) as potential medicines: O_2_^−•^ versus ^1^O_2_. *J. Am. Chem. Soc.* 125, 12803–12809. doi: 10.1021/ja0355574

Zhao, B., Bilski, P. J., He, Y.‐Y., Feng, L., and Chignell, C. F. (2008). Photo-induced reactive oxygen species generation by different water-soluble fullerenes (C_60_) and their cytotoxicity in human keratinocytes. *Photochem. Photobiol.* 84, 1215–1223. doi: 10.1111/j.1751-1097.2008.00333.x

Zhuang, Z., Dai, J., Yu, M., Li, J., Shen, P., Hu, R., et al. (2020). Type I photosensitizers based on phosphindole oxide for photodynamic therapy: apoptosis and autophagy induced by endoplasmic reticulum stress. *Chem. Sci.* 11, 3405–3417. doi: 10.1039/d0sc00785d
